# Advancing rehabilitation through health policy and systems research

**DOI:** 10.2471/BLT.22.289208

**Published:** 2022-11-01

**Authors:** Alarcos Cieza, Bente Mikkelsen, Abdul Ghaffar

**Affiliations:** a Department of Noncommunicable Diseases, World Health Organization, Avenue Appia 20, 1211 Geneva 27, Switzerland.; b Alliance for Health Policy and Systems Research, World Health Organization, Geneva, Switzerland.

The rehabilitation sector acknowledges that business as usual will not address the huge unmet need for rehabilitation. Globally, up to half of the 2.4 billion people who could benefit from rehabilitation are unable to receive it,^1^^,^^2^ thus impeding their chances of optimal functioning and improved health and well-being.

Evidence is needed that supports decisions about how to organize society and health systems to ensure that quality rehabilitation is scaled up and its access is equitable.^3^ Health policy and systems research seeks to produce this evidence.^4^

This theme issue of the *Bulletin of the World Health Organization* addresses the central question of how health systems can be organized to integrate rehabilitation. Although rehabilitation is by its nature integrative and an essential part of universal health coverage (UHC), in many countries it is still not a part of the continuum of care. Rehabilitation is rarely provided at all levels of care and in many settings continues to be viewed as a service exclusively for persons living with disabilities, delivered in specialized facilities and outside of the health system.^5^

The papers included in this theme issue look at how health systems can integrate rehabilitation into other health services, such as ear and hearing care,^6^ acute care,^7^ emergencies^8^ and the treatment of the post COVID-19 condition.^9^ Integration in primary care gets special attention^10^^–^^12^ because unless rehabilitation is provided and scaled up close to where people live, societies and health systems will not be able to meet the current global unmet need.^13^ This focus is in line with WHO’s current priority of providing health, by reorienting health systems towards primary health care as the foundation of UHC.^14^ This theme issue also provides evidence on specific health system concerns related to UHC, such as rehabilitation in health financing^15^ and the need for the rehabilitation workforce to be considered in workforce planning processes.^16^^,^^17^

Rehabilitation stakeholders will need to continue investigating relevant health policy and systems research questions.^18^ For example, is it necessary to have strong secondary and tertiary level rehabilitation before expanding it to primary care? Do policy strategies, such as those that link rehabilitation to pension and accident insurances, create inefficiencies and inequities? What are the most efficient procurement and supply chain management approaches for the availability of quality assistive products at the delivery point of rehabilitation?

Policy-makers involved in rehabilitation decision-making need to ensure that health systems become learning health systems^19^ for rehabilitation. Doing so involves the use of international health policy and systems research evidence and its integration with national data and expertise as well as adopting systems thinking^20^ to create knowledge that guides decision-making and implementation at the national level. Systems thinking helps to explain why specific systems outcomes – such as rehabilitation workers leaving the country after completing their training – have happened and not others.

The rehabilitation community and policy-makers should take advantage of opportunities to increase the use of health policy and systems research for rehabilitation. First, compared with a decade ago, the need for health policy and systems research evidence is now more widely acknowledged.

Second, WHO’s launch in 2017 of *Rehabilitation 2030: A call for action*^21^ urged stakeholders to recognize that rehabilitation is an essential health strategy that is part of UHC and embedded in health systems. The call for action has since been adopted by increasing numbers of stakeholders.

Third, more than 30 countries are implementing strategic plans for rehabilitation using WHO tools, and collaborating with development partners, civil society and service users. These countries are setting objectives to strengthen health systems for rehabilitation and executing actions to achieve these objectives. For example, Georgia is integrating rehabilitation into the benefit packages of care for UHC. Health policy and system research questions should be embedded in those endeavours.

The generation and use of health policy and system research evidence require targeted resources. More investment in health policy and system research for rehabilitation is needed. The United States Agency for International Development (USAID) made such an investment with a call for proposals for health policy and system research in rehabilitation in 2019 of 40 million United States dollars.^22^ We count on other agencies to follow. Advances in science and research are necessary to change business as usual, increase our understanding and open new possibilities. This theme issue supports the possibility of a world in which everyone who needs rehabilitation receives quality services for optimal functioning and improved health and well-being.
